# HSP27/Menin Expression as New Prognostic Serum Biomarkers of Prostate Cancer Aggressiveness Independent of PSA

**DOI:** 10.3390/cancers14194773

**Published:** 2022-09-29

**Authors:** Asma Bourefis, Hajira Berredjem, Omar Djeffal, Thi Khanh Le, Sophie Giusiano, Palma Rocchi

**Affiliations:** 1 Laboratory of Applied Biochemistry and Microbiology, Department of Biochemistry, Faculty of Sciences, Badji Mokhtar-Annaba University, Annaba 23000, Algeria; asma.bourefis@univ-annaba.org; 2 Private Medical Uro-Chirurgical Cabinet, Annaba 23000, Algeria; omar_djeffal@yahoo.fr; 3 Predictive Oncology Laboratory, Centre de Recherche en Cancérologie de Marseille, Inserm UMR 1068, CNRS UMR 7258, Institut Paoli-Calmettes, Aix-Marseille University, 27 Bd. Leï Roure, 13273 Marseille, France; khanh.le-thi@inserm.fr; 4 Department of Pathology, Hôpital Nord, Chemin des Bourrely, Aix Marseille University, CEDEX 20, 13915 Marseille, France; sophie.giusiano@ap-hm.fr

**Keywords:** prostate cancer, HSP27, Menin, Elisa, IHC, prognosis, tumor biomarkers

## Abstract

**Simple Summary:**

Cancer biomarkers are biomolecules synthesized by the tumor cell and have a role in early detection of cancer and determining its stage and progression. HSP27 and Menin are biomolecules involved in tumorigenesis and cancer progression; they are overexpressed in many cancers and could be useful as biomarkers. However, the overexpression of HSP27 and Menin in the serum of prostate cancer (PCa) patients and the correlation they have with their tissue expression, as well as their correlation with the clinical data, have not been explored yet. In this study, we first investigated the correlations between HSP27 and Menin in PCa patients, and then we examined the signification of HSP27/Menin in the diagnosis and prognosis of PCa using ROC analysis. Our results suggest that HSP27/Menin are dependent biomarkers in aggressive PCa and are significantly associated with poor prognosis; they could be used in the diagnosis and clinical decision making of individual PCa patients.

**Abstract:**

The screening of PCa is based on two tests, the total PSA test and the rectal examination. However, PSA is not specific for PCa stage confirmation, leading in false positive result and involving PCa over-diagnosis and over-treatment. HSP27 and Menin have been found to be overexpressed in a wide range of human cancers. Recent studies showed how HSP27 interacts with and stabilizes Menin to lead PCa progression and treatment resistance. The purpose of our study was to evaluate the correlation of HSP27 and Menin molecular expression, and their prognosis value in PCa with respect to clinicopathological features. Elisa was employed to measure serum HSP27 and Menin concentrations in 73 PCa patients and 80 healthy individuals. Immunohistochemistry (IHC) was used to determine HSP27 and Menin tissue expression in 57 tumors and 4 Benign Prostatic Hyperplasia (BPH) tissues. Serum HSP27 expression correlated with its tissue expression in all PCa patients, whereas serum Menin expression correlated only with tissue expression in aggressive PCa patients. Moreover, the results showed a positive correlation between HSP27 and Menin either in serum (r = 0.269; *p* = 0.021) or in tissue (r = 0.561; *p* < 0.0001). In aggressive PCa, serum expression of HSP27 and Menin was positively correlated (r = 0.664; R = 0.441; *p* = 0.001). The correlation between HSP27 and Menin expression in tissue was found only in patients with aggressive PCa (r = 0.606; R = 0.367; *p* = 0.004). Statistical analysis showed that the expression of both biomarkers was positively correlated with the hormone resistance or sensitivity, tumor aggressiveness, metastasis, Gleason Score, death and did not significantly correlate with age and PSA. Survival was illustrated by Kaplan–Meier curves; increased HSP27 and Menin expression correlated with shorter survival of PCa patients (*p* = 0.001 and *p* < 0.0001, respectively). Accuracy in predicting aggressiveness was quantified by the Area Under the Curve (AUC) of Receiver Operating Characteristic (ROC). We demonstrated that the combination of HSP27/Menin was statistically greater than PSA; it achieved an AUC of 0.824 (95% CI, 0.730–0.918; *p* < 0.0001). However, HSP27/Menin/PSA combination decreased the diagnostic value with an AUC of 0.569 (95% CI, 0.428–0.710; *p* = 0.645). Our work suggests the potential role of HSP27/Menin as diagnostic and prognostic biomarkers.

## 1. Introduction

In 2020, according to the World Health Organization (WHO), prostate cancer (PCa) is the third most commonly diagnosed malignancy, preceded only by lung and colorectal cancer. It is the most commonly diagnosed cancer in over 50% of countries in the world [1]. In Algeria, PCa is the fourth most common cancer; the incidence of PCa continues to increase, with 3597 new cases diagnosed, including 1635 deaths in 2020 [2]. The detection of PCa is worldwide based on the measurement of serum Prostate Specific Antigen (PSA), digital rectal examination, clinical stage and/or prostate volume [3]. However, PSA, which is used as tumor marker for the screening, diagnosis, monitoring, and prognosis of PCa, is a specific prostate activity marker but not specific for PCa [4]. This procedure leads to a high rate of needless biopsies and over-detection of insignificant PCa [5]. Frequently, the diagnosis must be followed by transrectal ultrasound guided biopsies [6]. Thus, there is a lack of reliable predictors of prognosis besides the Gleason Score and TNM stage. Hence, research focus on the developing of tests that are more specific for discriminating PCa stages and can enhance the selection of a diagnosis, treatment, prognosis evaluation, and disease monitoring. Only a small number of candidate molecular markers are currently used in clinical practice; however, many more have been reported over time and are being evaluated [7,8].

Heat Shock Proteins (HSPs) are a large family of highly conserved homologous chaperone proteins that are induced in response to environmental, physical and chemical stresses, and subsequently, they limit the consequences of damage and facilitate cellular recovery [9]. Some are constitutively expressed and associated with specific intracellular organelles, and others are rapidly induced in response to cellular stress [10]. It is constitutively expressed at low levels in the cytosol of most human cells [11], but upon certain stress conditions, it is redistributed into the nucleus or in the cytoskeleton [12,13]. HSPs protect normal cells against stress related injury but also increase the chances of survival of cancer cells by antiapoptotic activity, as an example. Furthermore, HSPs are often overexpressed and associated with increased tumorigenicity, metastatic potential, resistance to chemotherapy, and poor prognosis in many cancers [14]. Clinically, HSP27 is highly expressed in many cancers including breast [15], ovarian [13], gastric adenocarcinoma [16], lung cancer [17], colorectal cancer [18], prostate [19,20], and hepatocellular carcinoma [21]. In breast cancer, it has been shown that increased expression of HSP27 is a useful prognostic factor that correlates with significantly shorter disease survival, increased cell proliferation, metastases and resistance to chemotherapy [22]. In cervical neoplasia, association has been reported between overexpression of HSP27 and grade [23].

Previously, Cherif et al. [24] demonstrate that HSP27 is highly overexpressed in castration-resistant phenotype (CRPC) and acts as stress activated cytoprotective chaperone, supporting tumor cell survival and plasticity via various pathways [19,25]. Hence, HSP27 have clinical relevance as potential therapeutic targets in PCa patients. Moreover, the authors used a large scale proteomic approach which permitted them to identify Menin as a novel highly regulated HSP27 protein in PCa. 

Menin is a protein encoded by a Multiple Endocrine Neoplasia-type 1 gene (*MEN1*) that is classified as a gatekeeper tumor suppressor gene and which directly controls cell growth or death in selected tissues [26,27]. Interestingly, contrary to its role as a tumor suppressor, *MEN1* functions as an oncogenic partner in aggressive human acute leukemia and in hepatocellular carcinogenesis [28,29]. Patients carrying the mutated *MEN1* locus develop tumors, most frequently, in the parathyroid glands, pituitary glands, adrenal cortical section and islet cells of the pancreas. A number of non-endocrine tumors can also occur [27,30]. This protein is located predominantly in the nucleus but also in the cytoplasm and cell membrane during cell division [31]. The overexpression of Menin is often associated with the removal of the tumor, which confirms its role as a tumor suppressor [32]. Otherwise, it has an oncogenic function with poor prognosis in hepatocellular carcinoma [29], breast cancer [33] and PCa [34].

A combination of biomarkers such as HSP27 and Menin may help to improve the diagnosis, prognosis and overall therapeutical approach. The purpose of our study was to evaluate the molecular expression value of HSP27/Menin in PCa with respect to clinicopathological features. Furthermore, HSP27/Menin has been investigated as possible diagnostic and prognostic factors. We therefore sought to ascertain whether a correlation would be inter-observed between these two proteins using Principal Component Analysis (PCA), Factorial Correspondence Analysis (FAC) and Receiver Operating Curve (ROC) to evaluate for the first time, to our knowledge, the potential value of HSP27/Menin as a circulating biomarker.

## 2. Materials and Methods

### 2.1. Patient Population

This study was approved by the local Ethics Committee (number CEDUBMA-02-12/21) from University of Badji Mokhtar, Annaba, and was conducted according to the declaration of Helsinki; all patients and control subjects have provided written informed consent before enrolment. 

We prospectively recruited, between 2017 and 2019, a total of 153 subjects, including 73 men in the age range of 51–91 years (74.210 ± 9.217 years) with histologically confirmed PCa, and 80 healthy individuals in the age range of 29–80 years (61 ± 1.053 years) as control group. None of the participants had a family history of PCa. Patients were followed for 3 to 5 years. The diagnosis of PCa was based on serum PSA ≥ 4.0 ng/mL, presence of a palpable nodule on prostate digital rectal examination and histological parameters. Furthermore, all clinicopathological information from the patients with PCa was extracted from clinical records and is summarized in Table 1. 

The histopathological grade was classified into Gleason Scores [35]: Score 6 (N = 17); Score 7 = 3 + 4 (N = 7); Score 7 = 4 + 3 (N = 13); Score 8–10 (N = 20). In order to find the possible relationship between the studied biomarkers and clinical prognosis, patients were divided into three groups: non aggressive (Gleason Score ≤ 6; N = 17), moderately aggressive (Gleason Score = 7; N = 20) and aggressive (Gleason Score 8–10; N = 20). A total of 73 serums from PCa patients were sampled during the diagnostic or surgical act. Blood samples were collected in dry tubes then centrifuged at 1800 rpm/min for 15 min. Serum was recovered in aliquots and stored at −20 °C until further use. We also collected the matched biopsies from 73 related PCa patients and 4 Benign Prostatic Hyperplasia (BPH). Tissue samples (needle biopsies) collected at initial diagnosis and before surgery were fixed in formalin and embedded in paraffin then immediately stored at −20 °C.

Exclusion criteria for study participation were as follows: inability to actively consent to the study; patients undergoing hormone therapy or radiotherapy at the time of blood sample, because of the possible effects on protein expression and infections or other cancer diseases.

### 2.2. Quantification of Serum HSP27 and Menin

Human HSP27 and Menin expression were detected in serum by a commercially available Elisa Kits (Enzo ADI-EKS-500-96, Villeurbanne, Fr) and (My BioSource MBS-942430-96, San Diego, CA, USA), respectively, according to the manufacturer’s instructions. Briefly, 100 μL of standards and samples were dispensed into an immunoassay flat-bottomed 96-well plates and then incubated for 1 h, at 37 °C, for HSP27 and 2 h, at 37 °C, for Menin. Plates were washed, and then 100 µL of Anti-HSP27 and 100 µL of Biotin antibody were added into each well. The plates were incubated at room temperature for 1 h, at 37 °C. After another washing step, 6 times for HSP27 and 3 times for Menin, 100 µL of Horseradish–peroxidase conjugate were added to each well. The plate of HSP27 was incubated at room temperature for 30 min, and the plate of Menin was incubated at room temperature for 1 h. After a further washing step, 100 μL of TMB Substrate was applied to detect enzyme activity, and the plates were incubated at 37 °C for 15–30 min. The reaction was stopped with the addition of 100 μL (for HSP27) and 50 µL (for Menin) of stop solution into each well. It is important that all the blue color changes completely yellow in each well. Optical density was measured by a microplate reader (Sunrise, Tecan, 69003 Lyon, France) at 450 nm within 5 min. Finally, HSP27 and Menin concentrations were calculated after plotting the standard curves by professional software “CurveExpert”. Duplicates of each sample were measured.

### 2.3. Tissue HSP27 and Menin Expression

Only 57 out of 73 patients’ biopsies were included in the immunohistological analysis. We excluded biopsies which have been embedded in paraffin and presented no available result interpretation. Formalin-fixed paraffin blocks, 3–4 μm-thick sections were used for IHC. The immunohistochemical staining was examined by two pathologists, blinded to the evaluations.

For HSP27 detection, IHC staining was performed using Autostainer Link 48 from Dako (Agilent, Santa Clara, CA, USA). After deparaffinization, antigens retrieval was performed with Target Retrieval Solution pH9 (S2367, Agilent, Santa Clara, CA, USA) in PT Link (Agilent, Santa Clara, CA, USA). The slides were incubated at room temperature for 20 min with rabbit polyclonal antibody HSP27 (dilution 1:600, Enzo (ADI-SPA-803), Lausen, Suisse) detecting multiple epitopes. Then, an Envision Flex system (K8000, Agilent, Santa Clara, CA, USA) was used with DAB.

For Menin detection, IHC was carried out with mice monoclonal antibody Menin E9 sc390345 (1:100, Santa Cruz Biotechnology, D-69115 Heidelberg, Germany) on the Ventana Discovery XT (Roche, Paris, France). After deparaffinization, antigens retrieval was performed with Cell Conditioning Solution CC1 (Tris-EDTA-based buffer pH 7.8, ref. 950-124, Roche). The primary antibody was incubated for 6 h, at 37 °C, then the secondary antibody, OmniMap anti-Mouse HRP Detection Kit (ref. 760-4310), was used and followed by chromogen DAB. Finally, the counter staining was performed with hematoxylin and slides were cleaned, dehydrated and cover-slipped with permanent mounting media.

The specificity of antibodies has been validated for positive and negative control by the ICEP team prior to their use in IHC. Negative and positive controls were included in each batch of IHC. Sections of skin known to express high levels of HSP27 and sections of breast and prostate cancer who express high levels of Menin was included as positive control, while control without primary antibody was systematically included to rule out non-specific staining. Images were captured using a microscope Nikon ECLIPSE Ni.

The intensity of cytoplasmic or nuclear immunostainings as well as the proportion of stained cells were used to microscopically evaluate the expression of HSP27 and Menin. Scores for the staining intensity were ranged from 0 (negative) to 1 (weak staining), 2 (moderate staining), or 3 (strong staining). The percentage of positive tumor cells was scored as 0 (<1%), 1 (1–25%), 2 (25–50%), 3 (>50%). The intensity and percentage of marked cells was evaluated in a semiquantitative manner by calculating the Medium Quick Score (MQS). Thus, a MQS from 0 to 300 was obtained. 

### 2.4. Statistical Analysis 

The descriptive analysis and data were evaluated statistically using IBM SPSS Statistics version 25 (SPSS Inc., Chicago, IL, USA) and GraphPad Prism 8 software (GraphPad Software Inc., LA Jolla, CA, USA). The one-way analysis of variance (ANOVA) was used to compare parametric variables and expressed as mean ± standard error (SE). The Pearson correlation coefficient was calculated as a measure of linear association between continuous variables, including HSP27 and Menin in serum and tissue. The overall global survival analysis was performed by the Kaplan–Meier product limit method. Receiver Operating Curve (ROC) was performed to evaluate the potential value of HSP27 and Menin as circulating biomarkers in comparison with PSA. The Area Under the Curve (AUC) represented the diagnostic performance of the studied molecules.

Principal Component Analysis (PCA) and Factorial Correspondence Analysis (FCA), both descriptive multivariate statistical analysis methods for visualization and reduction in data set dimension, were performed with IBM SPSS version 25 software (SPSS, Chicago, IL, USA). In all comparisons, *p*-value < 0.05 was considered statistically significant.

## 3. Results

### 3.1. Patient Characteristics

The baseline clinicopathologic characteristics of the participants enrolled in this study are presented in Table 1. The study cohort included 153 men, among whom were 73 PCa patients and 80 controls. There were significant differences in age (*p* = 0.018). There were 66 (90.41%) participants with a PSA level > 10 ng/mL and 7 (9.59%) with a PSA level ≤ 10 ng/mL. During the follow-up, 29 (39.72%) men developed hormone resistance and 15 (20.54%) men died of PCa. Hence, with increasing age at diagnosis, cancer characteristics generally worsened more among men. 

### 3.2. Quantification of Serum HSP27 and Menin

**Figure 1:**
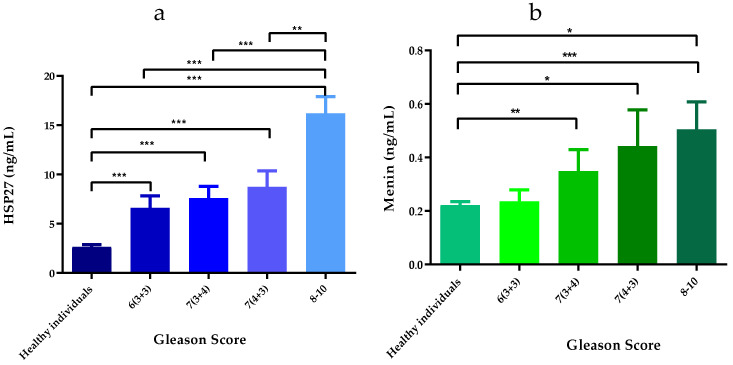
Serum HSP27 and Menin concentrations. (**a**) HSP27 expression in PCa patients according to the Gleason Score; (**b**) Menin expression in PCa patients according to the Gleason Score. Significant difference between different patients’ groups: * *p*-value ≤ 0.05, ** *p*-value ≤ 0.01, *** *p*-value ≤ 0.001.

The expression levels of HSP27 in PCa patients serum were significantly elevated (9.883 ± 0.853 ng/mL) when compared with that of healthy controls (2.657 ± 0.214 ng/mL) (*p* < 0.0001). The mean concentrations of HSP27 in PCa patients according to the Gleason Score were: (Score 6(3 + 3): (6.614 ± 1.202 ng/mL; *p* < 0.001)), (Score 7(3 + 4): (7.604 ± 1.205 ng/mL; *p* < 0.001)), (Score 7(4 + 3): (8.741 ± 1.630 ng/mL; *p* < 0.0001)) and (Score 8–10: (16.208 ± 1.695 ng/mL; *p* < 0.0001)). There were statistically significant differences in the HSP27 expression between the healthy individuals and the different Gleason scores among the PCa patients (Figure 1a). HSP27 level in aggressive PCa was 6.10-fold higher than that found in healthy controls.

Menin is overexpressed in PCa patients (0.375 ± 0.045 ng/mL) compared to healthy controls (0.221 ± 0.013 ng/mL) (*p* < 0.0001). The mean concentrations of Menin according to Gleason Score were: Score 6(3 + 3): (0.236 ± 0.043 ng/mL; *p* > 0.05), Score 7(3 + 4): (0.349 ± 0.079 ng/mL; *p* > 0.05), Score 7(4 + 3): (0.442 ± 0.135 ng/mL; *p* < 0.05) and Scores 8–10: (0.505 ± 0.102 ng/mL; *p* < 0.0001). There were statistically significant differences in the Menin expression between the healthy individuals and Gleason scores 7 and 8–10 among the PCa patients (Figure 1b). Menin level in aggressive PCa was 2.28-fold higher than that found in healthy controls.

Our results suggest that HSP27 and Menin could be useful biomarkers for predicting prognosis at level threshold between 6.614 and 16.208 ng/mL for HSP27 and 0.236 and 0.505 ng/mL for Menin.

### 3.3. Tissue HSP27 and Menin Expression

**Figure 2:**
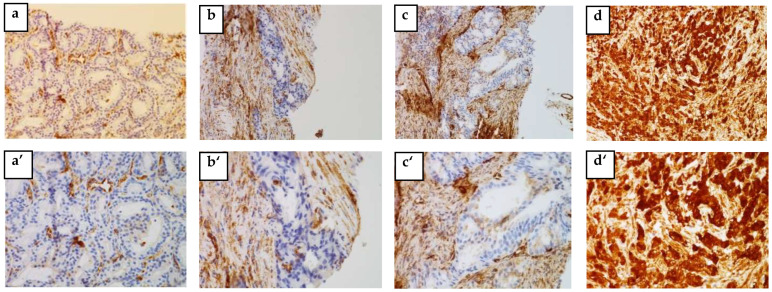
HSP27 expression is associated with aggressive human PCa. Representative tissue sections of PCa tumors from needle biopsies were stained for IHC specific for HSP27. (**a**,**a’**) BPH, (**b**,**b’**) weakly positive (non-aggressive PCa), (**c**,**c’**) moderately positive (moderate PCa) and (**d**,**d’**) strongly positive (aggressive PCa). (**a**–**d**) ×200 and (**a’**–**d’**) ×400 magnification.

**Figure 3:**
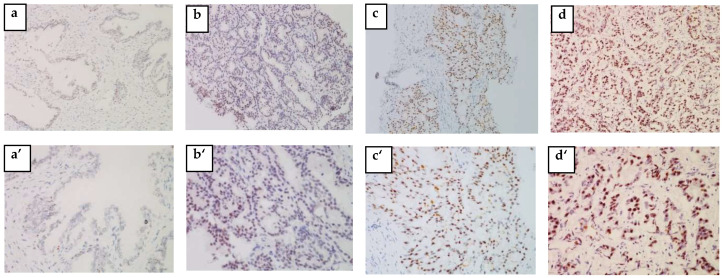
Menin expression is associated with aggressive human PCa. Representative tissue sections of PCa tumors from needle biopsies were stained for IHC specific for Menin. (**a**,**a’**) BPH, (**b**,**b’**) weakly positive (non-aggressive PCa), (**c**,**c’**) moderately positive (moderate PCa) and (**d**,**d’**) strongly positive (aggressive PCa). (**a**–**d**) ×200 and (**a’**–**d’**) ×400 magnification.

The IHC analysis of 57 out of 73 blocks of human PCa tissue permitted us to identify the cytoplasmic localization of HSP27 and the nuclear localization of Menin. Figure 2 and Figure 3 clearly illustrate the expression of HSP27 and Menin according to the aggressiveness of the tumor. HSP27 and Menin expression was stronger in PCa than in BPH prostate tissue.

The MQS of HSP27 was significantly higher (182.543 ± 9.063) in PCa tissues than in control BPH ones (16.750 ± 6.485) (*p* < 0.0001). According to the Gleason Score, the MQS of HSP27 in PCa patients was: (Score 6(3 + 3): (153.676 ± 22.090; *p* < 0.001)), (Score 7(3 + 4): (167.857 ± 27.257; *p* < 0.001)), (Score 7(4 + 3): (178.269 ± 17.365; *p* < 0.0001)) and (Scores 8–10: (215.00 ± 6.123; *p* < 0.0001)).

Menin MQS in PCa patients was significantly higher (168.236 ± 9.042) than in BPH (75 ± 53.033) (*p* < 0.0001). Nuclear Menin immunoreactivity was present in all PCa associated tissues. The MQS of Menin was significantly higher in Scores 8–10 (202.500 ± 7.884; *p* < 0.001) than in Score 6(3 + 3): (115.558 ± 19.423; *p* > 0.05), Score 7(3 + 4): (176.785 ± 26.785; *p* < 0.05) and Score 7(4 + 3): (179.807 ± 14.261; *p* < 0.05).

### 3.4. Correlation between Serum and Tissue HSP27 and Menin Expression

The linear correlation coefficient showed that the expression of HSP27 in the serum of patients with non-aggressive PCa is significantly positively correlated with its expression in tissue (r = 0.541; R = 0.292; *p* = 0.024). In moderately aggressive PCa, the correlation of serum HSP27 with its expression in tissue is positively significant (r = 0.654; R = 0.428; *p* = 0.001). In aggressive PCa, there is a significant and positive correlation between the expression of HSP27 in serum and tissue (r = 0.445; R = 0.198; *p* < 0.05).

There was no statistically significant difference in non-aggressive and moderately aggressive PCa between Menin expression in serum and tissue, (r = −0.328; R = 0.108; *p* = 0.197) and (r = 0.076; R = 0.005; *p* = 0.749), respectively. However, in aggressive PCa, Menin serum expression was significantly positively correlated with its expression in tissue (r = 0.530; R = 0.281; *p* = 0.01).

### 3.5. Correlation between HSP27 and Menin

We performed univariate analysis using Pearson test to investigate whether our biomarkers were clinically independent prognostic factors for PCa patients. Results showed that HSP27 and Menin in healthy control were neither correlated in the serum (r = −0.125; R = 0.015; *p* = 0.266) nor in the tissue (r = 0.500; R = 0.258; *p* = 0.49).

**Figure 4:**
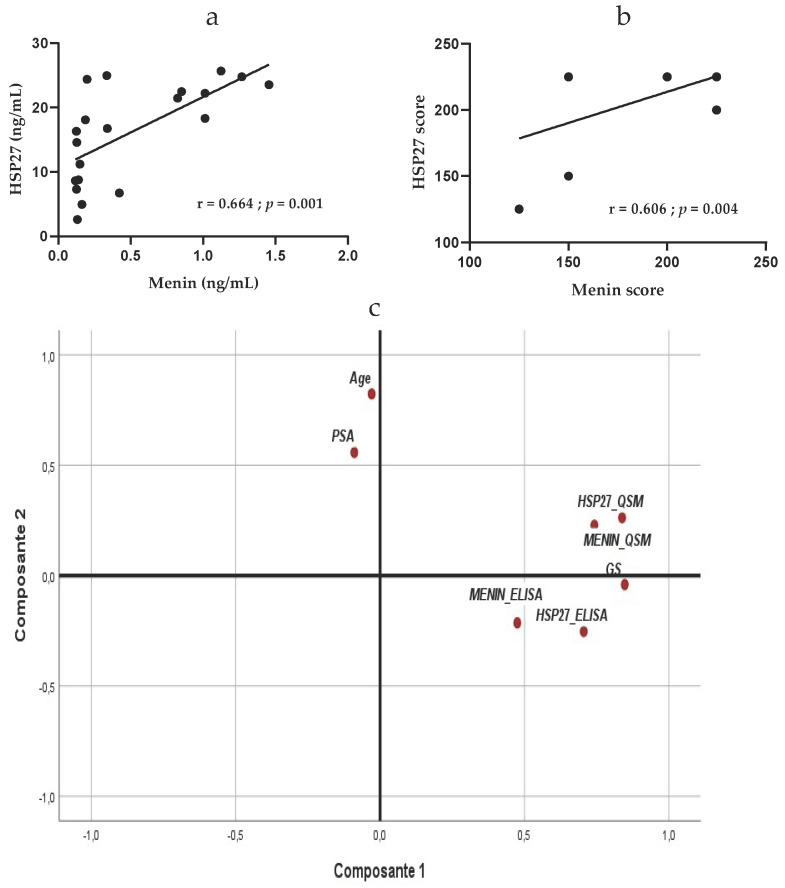
(**a**) Correlation between HSP27 and Menin serum expression in PCa aggressive patients; (**b**) Correlation between HSP27 and Menin tissue expression in PCa aggressive patients; (**c**) PCA of biological parameters (HSP27_QSM, MENIN_QSM: expression of HSP27 and Menin in tissue; HSP27_ELISA, MENIN_ELISA: expression of HSP27 and Menin in serum).

We did not observe a linear correlation between HSP27 and Menin levels in serum of patients with non-aggressive PCa (r = −0.108; R = 0.011; *p* = 0.677)**;** contrariwise, there was a significant inverse correlation between HSP27 and Menin in serum of patients with moderately aggressive PCa (r = −0.497; R = 0.247; *p* = 0.025)**.** In aggressive PCa, HSP27 expression is significantly positively correlated with Menin expression in serum (r = 0.664; R = 0.441; *p* = 0.001) (Figure 4a).

The correlation between HSP27 and Menin expression in tissue showed that these biomarkers are not correlated in non-aggressive and moderately aggressive, PCa (r = 0.143; R = 0.020; *p* = 0.582) and (r = 0.245; R = 0.060; *p* = 0.294), respectively, but are significantly positively correlated in tissue of patients with aggressive PCa (r = 0.606; R = 0.367; *p* = 0.004) (Figure 4b). These findings suggest that HSP27 and Menin might be dependent biomolecules for the diagnosis and prognostic of aggressive PCa.

Multivariate analysis using PCA in all PCa patients showed a positive correlation between HSP27 and Menin either in serum (r = 0.269; *p* = 0.021) or in tissue (r = 0.561; *p* < 0.0001). In the serum, HSP27 and Menin were positively correlated with their tissue expression (r = 0.522, *p* < 0.0001) and (r = 0.288; *p* = 0.015), respectively. Significant correlation was obtained between HSP27 (r = 0.452; *p* < 0.001) and Menin (r = 0.335; *p* < 0.001) for Gleason Score; however, no association was found between HSP27 and Menin with age, (r = −0.170; *p* = 0.103) and (r = −0.087; *p* = 0.261), and with PSA, (r = −0.084; *p* = 0.267) and (r = 0.005; *p* = 0.486), respectively.

In the tissue, Gleason Score was correlated with the expression of HSP27 (r = 0.687; *p* < 0.0001) and Menin (r = 0.506; *p* < 0.001); no correlation was obtained with age (r = −0.057; *p* = 0.337) and PSA (r = −0.128; *p* = 0.185).

Based on PCA results, PSA did not correlate with Gleason Score (r = −0.128; *p* = 0.185), HSP27, either in serum (r = −0.083; *p* = 0.269) or tissue (r = 0.039; *p* = 0.387), Menin, either in serum (r = 0.005; *p* = 0.486) or tissue (r = −0.022; *p* = 0.436), Age (r = 0.113; *p* = 0.202) and progression of disease (Figure 4c). 

### 3.6. FCA of Clinical Parameters of Studied Population

**Figure 5:**
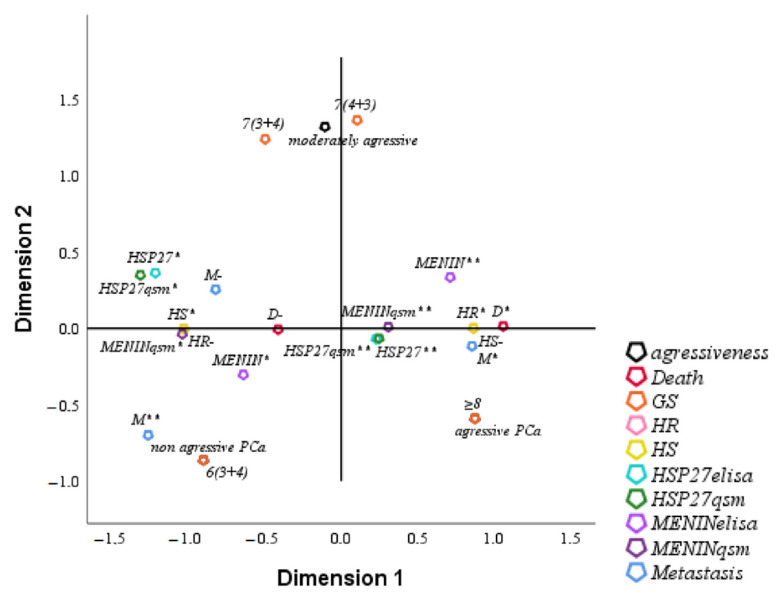
FCA of clinical parameters. M*: Metastasis, M-: No metastasis, M**: Recovery, D*: death, D-: Survival, HSP27**: over expression of HSP27 in serum, HSP27qsm**: over expression of HSP27 in tissue, Menin**: over expression of Menin in serum, Menin qsm**: over expression of Menin in tissue, HR*: Hormone resistance, HR^-^: No hormone resistance, HS*: Hormone sensitivity, HS^-^: No hormone sensitivity.

FCA is an extension of PCA to analyze the association between two or more qualitative variables based on the average proper value of the coefficient alpha of Cronbach ≥0.7 (0.814 in our study) and on the total proper value of the dimensions ≥50% (75% in our study). The analysis of HSP27 by multifactorial FCA showed a positive correlation with Menin either in the serum or in tissue. The overexpression of HSP27 and Menin in serum and tissue was correlated with HR, tumor aggressiveness, metastasis, Gleason Score and death. Low levels of HSP27 and Menin were correlated with HS, non-aggressive tumor, Gleason Score and survival (Figure 5).

### 3.7. ROC and Survival Analysis

**Figure 6:**
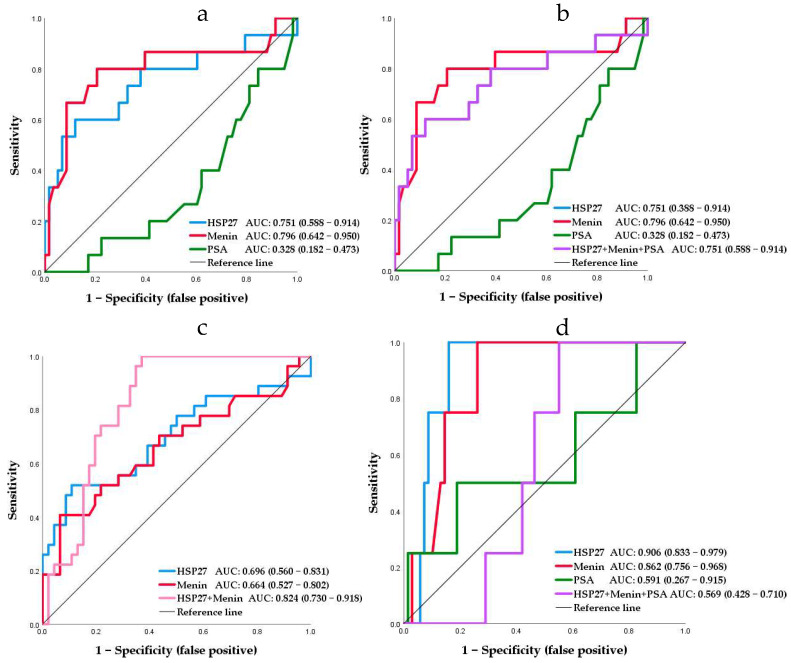
ROC analysis. (**a**,**b**) HSP27, Menin, PSA compared to death; (**c**,**d**) HSP27, Menin, PSA compared to aggressivity. HSP27: heat shock protein, PSA: prostate specific antigen, ROC: receiver operating characteristic, AUC: area under curve.

To further understand the diagnostic and prognostic role of HSP27, Menin and PSA in PCa, ROC analysis was performed. The ROC curves show the relationship between true positive and false-positive rates as well as the AUC. HSP27, Menin and PSA with AUC values higher or equal than 0.7 were validated. Quantification of predicting death was assessed by AUC. ROC curve could derive optimal statistically significant cut-offs that represent the most discriminative value, associated with the best sensitivity/specificity couple: (i) 9.310 ng/mL for serum HSP27 with a sensitivity of 80% but at a low specificity of 37%, (ii) 0.304 ng/mL for serum Menin with a sensitivity of 80% but at a low specificity of 20%, and (iii) 21.655 ng/mL for serum PSA with a sensitivity of 66% and specificity of 81%. The higher cut-off value of biomarkers HSP27 and Menin would result in an increase in true positive but PSA would result in an increase in false positive. However, the cut-offs value of serum HSP27 and Menin can be used for identifying patients with a high risk of death. As depicted in Figure 6a, the ROC curve analysis for death showed that PSA had the lowest AUC value 0.328 (95% CI: 0.182–0.473, *p* = 0.041); however, the AUC value for HSP27 and Menin serum concentration was 0.751 (95% CI: 0.588–0.914, *p* = 0.003) and 0.796 (95% CI: 0.642–0.950, *p* < 0.0001), respectively. We applied the optimal cut-offs to the validation of combined biomarkers HSP27, Menin and PSA (Figure 6b) and achieved the same AUC as that for HSP27.

The accuracy in predicting aggressiveness was also quantified by the ROC curve. The results showed the following: (i) 75% sensitivity and 13% specificity at the optimal cut-off value 17.985 ng/mL for serum HSP27, (ii) 75% sensitivity and 15% specificity at the optimal cut-off value 0.636 ng/mL for serum Menin, and (iii) 26% sensitivity and 60% specificity at the optimal cut-off value 60 ng/mL for serum PSA. Both HSP27 and Menin cut-offs were increased two-fold in comparison to cut-offs obtained with ROC predicting death. The highest AUC of 0.906 (95% CI: 0.833–0.979; *p* = 0.007) was obtained for HSP27, an intermediate AUC of 0.862 (95% CI: 0.756–0.968; *p* = 0.015) was obtained for Menin, and the lowest AUC of 0.591 (95% CI: 0.267–0.915; *p* = 0.545) was obtained for PSA.

The combination of HSP27/Menin was statistically greater than PSA and significantly improved the diagnostic and prognostic values; it had the best performance with an AUC of 0.824 (95% CI, 0.730–0.918; *p* < 0.0001) (Figure 6c). However, HSP27/Menin/PSA combination decreased the diagnostic value with an AUC of 0.569 (95% CI, 0.428–0.710; *p* = 0.645) (Figure 6d). ROC analysis results suggest that HSP27 and Menin are associated with death and aggressiveness but not PSA. Hence, HSP27 and Menin could be useful biomarkers to highlight at values greater than or equal to 9.310 and 0.304 ng/mL, respectively; however, PSA cannot be used as a prognostic factor in PCa patients.

**Figure 7:**
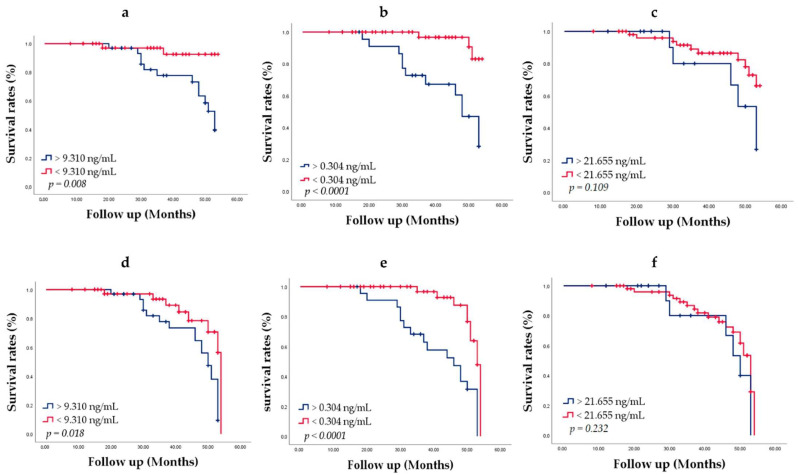
Overall survival according to death: impact of (**a**) HSP27, (**b**) Menin and (**c**) PSA on concentrations (ng/mL) PCa-specific survival. Overall survival according to metastasis: impact of (**d**) HSP27, (**e**) Menin and (**f**) PSA concentrations (ng/mL) on PCa-specific survival. Cut-offs HSP27, Menin and PSA were 9.310, 0.304 and 21.655 ng/mL, respectively. *p*-values were calculated by using the Log Rank test.

To evaluate the chance of survival and risk of death, we used the Kaplan–Meier method of survival analysis. Men were followed up for 3–5 years from the date of diagnosis. Patient groups were defined by the HSP27, Menin and PSA cut-offs determined through ROC analysis. Patients alive at the last follow-up were censored, since death was considered an event. During the follow-up of 73 patients, PCa metastasis was found in 29 (39.72%) patients; 15 (20.54%) men died of PCa. With increasing age at diagnosis, men had more comorbidity. Survival curves are shown in Figure 7.

The median ages of patients with low HSP27 and Menin concentration were higher than those of patients with high HSP27 and Menin concentration. Kaplan–Meier curve analysis related to death showed that the overall survival of patients with HSP27 < 9.310 ng/mL (94.9%) was significantly better than that of patients with HSP27 > 9.310 ng/mL (61.8%; Log Rank *p* = 0.008) (Figure 7a). The median survival of HSP27 > 9.310 ng/mL patients was 53.000 ± 1.805 (95% CI, 49.462–56.538). PCa survival diminished markedly in people with Menin value > 0.304 ng/mL (50.0%) compared to Menin value < 0.304 ng/mL (93.9%; Log Rank *p* < 0.0001) (Figure 7b). The median survival of Menin > 0.304 ng/mL patients was 48.000 ± 2.629 (95% CI, 42.848–53.152). Lower survival rates were obtained for PSA > 21.655 ng/mL (68.86%) than PSA < 21.655 ng/mL (82.5%) (Log Rank *p* = 0.109) (Figure 7c); no significant difference was observed. The median survival of PSA < 21.655 ng/mL patients was 53.000 ± 3.640 (95% CI, 45.866–60.134).

These results revealed that elevated HSP27 and Menin expression levels was associated with poorer prognosis in PCa patients and might predict cancer related death. However, PSA expression was not significantly associated with PCa survival (Log Rank *p* = 0.055).

Recurrence rates of the patients according to PCa metastasis status HSP27, Menin and PSA expression were also evaluated by the Kaplan–Meier method, which achieved for HSP27 > 9.310 ng/mL an overall survival-free metastasis of 38.2% (Log Rank *p* = 0.018) (Figure 7d) with a median survival of 50.000 ± 1.688 (95% CI, 46.691–53.309). The overall survival-free metastasis for Menin > 0.304 ng/mL was statistically significant (20.8%; Log Rank *p* < 0.0001) with a median survival of 46.000 ± 5.296 (95% CI, 35.620–56.380). Considering Menin < 0.304 ng/mL, the overall survival-free metastasis was statistically significant (79.6%; Log Rank *p* < 0.0001) (Figure 7e) with a median survival of 53.000 ± 0.609 (95% CI, 51.806–54.194). There was no significant difference for PSA. Lower survival rates were obtained for PSA > 21.655 ng/mL (56.3%) with a median survival of 50.000 ± 2.627 (95% CI, 44.852–55.148) than PSA < 21.655 ng/mL (61.4%) (Log Rank *p* = 0.232) with a median survival of 53.000 ± 0.870 (95% CI, 51.294–54.706) (Figure 7f).

## 4. Discussion

Despite the development of PCa screening in the late 1980s based on the measurement of PSA in serum, current PCa diagnosis and therapy approaches still have a number of limitations. Aside from Gleason score and TNM stage, there are few accurate markers of prognosis. The ability to differentiate between indolent and clinically aggressive PCa remains a challenge. Currently, when PSA levels exceed 4.0 ng/mL, most men undergo needle biopsy, but only about half of these biopsies results in a PCa diagnosis [36]. Subsequently, the difficulty to discern aggressive cancer from non-aggressive cancer contributes to the problem of over-treating men with non-aggressive disease. Accurate PCa assessment is crucial for selecting appropriate treatment in men with aggressive prostate cancer, while avoiding the disadvantages of aggressive treatment in men with non-aggressive tumors and improving quality of life [37]. PSA has merits as a screening biomarker with a positive impact on PCa diagnosis; however, PSA is unable to detect aggressive PCa, which has clinical implications for treatment. As a result, the wide use of PSA to detect PCa has raised concerns regarding over-diagnosis and over-therapy of low grade non-clinically significant disease [38,39]. This leads to the question of how to deal with PCa patients in an individualized therapeutic setting. Research is ongoing in the hopes of generating tests that are not only sensitive and specific for PCa, but also improve treatment choice, prognosis evaluation, and disease monitoring [7,10].

Biomarkers play a significant role in the diagnosis and prognosis of a majority of diseases such as cancers in today’s clinical field, and may provide guidance on therapy strategy and follow-up [40]. The main concern in our present study was an effort to investigate the diagnosis and prognosis value of HSP27 and Menin in comparison with PSA in PCa, depending on the aggressiveness status within the tumor. We believe that the present work is the first case–control study that proves that HSP27 and Menin are dependent predictors and potential discriminators of advanced PCa patients. Moreover, our results support that HSP27/Menin combination could represent a potential therapeutic opportunity for PCa treatment and improve patients survival. To the best of our knowledge, this is the first report that has determined the serum concentration of human Menin. In fact, in the literature, the evaluation of Menin expression has been carried out using techniques including IHC, Western blotting, and RT-qPCR [24,41]. Moreover, this study investigates, for the first time, the value of HSP27/Menin/PSA combination for the evaluation of PCa diagnosis and prognosis.

An association between over- or underexpression of HSPs and prognosis has been seen for some cancers, but the prognostic impact of HSPs expression varies between tumor types, either resulting in prolonged or shortened survival [8]. Concerning Menin, its overexpression is associated with removal of the tumor, which enhances its role of tumor suppressor [32,42], but it has also an oncogenic function. In fact, Menin have a poor prognosis in hepatocellular carcinoma, breast cancer [21,42] and prostate cancer [24].

Consistent with previous studies [8,20,33,43], our data underline the overexpression of HSP27 and Menin. More interestingly, our study demonstrates for the first time the correlation between tissue and serum HSP27 and Menin expression in PCa. In fact, the HSP27 serum concentrations were correlated positively with HSP27 expression in PCa tissues of all PCa patients. The Menin serum concentrations were correlated positively with Menin expression in aggressive PCa tissues. Overexpression of Menin was a significant prognostic factor for poor prognosis in aggressive PCa (r = 0.530; R = 0.281; *p* = 0.01). In addition, HSP27 and Menin were significantly correlated with early and advanced PCa showing a stage-dependent increase. Furthermore, HSP27 and Menin serum overexpression were significantly indicative for aggressive PCa, and could act as predictors of prognosis in PCa. Otherwise, we showed that HSP27 and Menin are dependent markers in aggressive cancer, which means that Menin leaves the nucleus to the cytoplasm, binds to HSP27 and then leaves the cell to the serum. Further research needs to be conducted to understand how Menin could leave the nucleus and bind with HSP27 in the cytoplasm and end up outside the cell in the serum.

To further explore and improve the diagnostic and prognostic efficiency of the studied biomarkers, we focused on a combined HSP27/Menin plus PSA model. To this end, we selected for HSP27, Menin and PSA cut-offs of 9.310, 0.304 and 21.655 ng/mL, respectively, defined by the ROC analysis. ROC curves are a useful tool for comparing the diagnostic ability of two or more screening tests or for assessing the predictive ability of two or more biomarkers for the same disease. The area under an ROC curve provides a measure of discrimination and allows investigators to compare the performance of two or more diagnostic tests. In general, the test with the higher AUC may be considered better [44]. Our results showed that ROC analysis provided an overall net benefit when compared with the correlation analysis. This was further demonstrated in the AUC area achievement, which clearly revealed that high HSP27/Menin expression predicted a worse prognosis, while low HSP27/Menin expression was correlated with better survival, indicating that these biomolecules are prone to serve as possible diagnostic and prognostic markers for PCa progression. Otherwise, this test could discriminate non-aggressive from aggressive PCa independent of PSA. HSP27/Menin were better able to distinguish aggressive (≥8) from clinically indolent PCa. In fact, cut-offs in the aggressive PCa were increased two-fold in comparison to that of non-aggressive PCa. This result corroborates data obtained in the correlation analysis showing a positive correlation of HSP27 and Menin. When compared with PSA, the studied markers HSP27 and Menin had a significantly higher diagnostic performance than PSA alone for patients with elevated PSA levels. Moreover, the combination of HSP27/Menin revealed a better AUC (0.824) than that of HSP27/Menin/PSA (0.569) and PSA (0.591). Previous report showed that in non-small cell lung cancer (NSCLC), HSP27 serum mean levels were 5.364 ± 2.679 ng/mL in patients with advanced stage NSCLC; AUC was 0.870 (95% CI; 0.817–0.923; *p* < 0.0001) [45]. In severely injured patients, Haider et al. [46] found significantly increased HSP27 serum concentrations in deceased patients (9.261 ± 2.817 ng/mL; *p* < 0.01) with AUC equal to 0.911 (95% CI: 0.818–1.000; *p* < 0.0001). Furthermore, Wang et al. [47] depicted an AUC of 0.720 (95% CI = 0.64–0.80; *p* = 0.0065) for predicting subclinical atherosclerosis in type 2 diabetes; the optimal cut-off value of HSP27 was 7.16 ng/mL.

Our PSA results corroborate previous studies [38,48] that showed for PSA ≥ 4 ng/mL an AUC of 0.560 and 0.570, respectively. For PCa with Gleason score ≥ 7, Kim et al. [37] obtained for PSA an AUC of 0.58. Moreover, van Gils et al. [48] determined a PSA cut-off value of 58 ng/mL (sensitivity 48% and specificity 62%). A recent study [49] reported that the percentage of patients with GS ≥ 8 or metastases increased as PSA levels increased up to approximately 70 ng/mL; however, PSA cannot be used as a prognostic factor in patients with PSA levels ≥ 70 ng/mL. Hence, PSA, which is widely used for PCa diagnosis, is not an indicator of malignancy it is a biological parameter that can only tell the presence of a problem. Previous study has shown that PSA testing has low sensitivity when the PSA cut-off value is set at ≥4 ng/mL, and the diagnostic rate among patients with a PSA level of 4–10 ng/mL was much lower [6]. PSA alone is no longer useful as a prognostic marker. We strongly believe that our findings may enable clinicians in determining whether prostate biopsies should be performed, particularly for patients who had no other symptoms other than increased PSA.

In our cohort, the risk of prostate cancer death increased step wise with increasing HSP27 and Menin concentration. Hence, increased expression of HSP27 and Menin correlates with poor patient survival. Old age at diagnosis was also associated with higher risk of PCa death. Patients with HSP27 > 9.310 ng/mL could develop a metastasis within 50 months, with an overall survival percentage of 60.3%, while patients with HSP27 < 9.310 ng/mL did not develop a metastasis until the end of our study (79.5%; Log Rank *p* = 0.018). Otherwise, we noticed that patients with Menin levels > 0.304 ng/mL could develop metastasis in a shorter time (46.000 ± 5.296) with an overall survival-free metastasis corresponding to 20.8% compared to that of Menin < 0.304 ng/mL presenting an overall survival-free metastasis equivalent to 97.6%. Bechis et al. [50] observed a poorer prognosis with advancing age at diagnosis in males treated for localized PCa with radical prostatectomy, radiation, or primary androgen deprivation therapy. The increased risk of upstaging and upgrading upon radical prostatectomy has also been linked to older age at diagnosis, showing that PCa in older men is more aggressive [51].

When examined in relation to clinicopathological features, data of this study suggests that the level expression of HSP27 and Menin in patients with PCa is not correlated with age and PSA, but is correlated with HR, HS, Metastasis, Gleason Score, tumor aggressiveness, tumor progression and death. Moreover, in patients with non-aggressive PCa, HSP27 and Menin have a good prognosis, whereas in patients with aggressive PCa, these biomarkers have a poor prognosis. Overall, our data therefore highlight the fact that HSP27/Menin are dependent markers in serum; so, it is a question of inhibiting one of the two markers in order to block the expression of the other and eventually stop the disease. In this perspective, Palmas’ team suggests that Menin interacts with HSP27 and its inhibition with ASO blocs the expression of HSP27 that protects Menin from degradation. Menin is overexpressed in high-grade PCa and CRPC plays a role in PCa therapy resistance by activating the PI3K/AKT pathway. Chemotherapeutic sensitivity is recovered by Menin inhibition. Menin is silenced by ASO technology, which also decreases tumor growth and CRPC cell proliferation while restoring chemotherapeutic sensitivity. These findings support the idea of targeting Menin to enhance the therapeutic effects for CRPC patients [24]. A second-generation antisense oligonucleotide (ASO) called OGX-427 (Apatorsen), which Rocchi et al. [19,52] created to target the HSP27 protein’s overexpressed mRNA in the CRPC, is currently undergoing a Phase II clinical trial in the United States and Canada.

In addition, HSP27 and Menin can serve as liquid biopsies that could be a guide in the choice of personalized therapy as well as useful in the definition of inclusion criteria and stratification of patients in other clinical studies. The PSA assay has little potential to detect PCa at an early stage, the biomarkers HSP27 and Menin have the potential not only to detect the disease at an early stage to reduce cancer mortality and increase patient survival but also to differentiate true and false positive PCa screens. Based on our results, we find highlighted interest of the liquid biopsy in the detection of HSP27 and Menin high concentrations; these biomarkers would be useful as a screening tool for PCa and would differentiate patients with cancer from those with benign or healthy lesions. HSP27/Menin are useful for monitoring patients during treatment and measuring each patient’s response to treatment.

The population-based sample and inclusion of all risk factors make this study effective. For a period of three to five years, we could follow the PCa cases. However, there are certain limitations on this study. It was carried out in a single-center investigation and requires validation with larger sample sizes at more independent research centers. Second, this study included only patients who underwent biopsy and had results available in our institutional patient records. We excluded patients who underwent radical prostatectomy. In addition, we did not inspect the percentage of tumor involvement in each biopsy core and tumor size.

## 5. Conclusions

This study demonstrates that HSP27 and Menin achieved superior performance for patient level detection of higher grade PCa than that of conventional PSA. Subsequently, the overexpression of HSP27 and Menin plays an important role in PCa patient’s survival. Elisa measurement of serum HSP27 and Menin concentrations may be useful for screening for PCa stage and offer a prospective use for these proteins as a prognostic molecular marker. Our results are biologically significant not only for investigating the onset and progression of PCa, but also for clinically detecting individuals who require prompt aggressive treatment to control the potentially lethal disease. Of utmost interest to us was the finding that our results also suggest that the diagnostic performance of combined HSP27/Menin to predict the presence and aggressiveness of PCa performed better than either PSA alone or HSP27/Menin/PSA combination. The data reported here may have important clinical relevance, having the potential to impact treatment decisions for patients. Further multicenter prospective studies are needed to support these results.

## Figures and Tables

**Table 1:** Clinicopathologic characteristics of individuals with and without PCa.

Characteristics	Cases (*n* = 73) N (%)	Controls (*n* = 80) N (%)	*p* Value
Age (years)			
mean ± SE	73.26 ± 10.13	70.53 ± 8.38	0.018
**PSA at diagnosis (ng/mL)**			
mean ± SE	80.72 ± 24.34	3.54 ± 0.72	
≤10	7 (9.59)		<0.0001
>10	66 (90.41)		
**Gleason score**			
≤6 (Low)	23 (31.51)		
=7 intermediate	30 (41.09)		
≥8 (High)	20 (27.40)		
**Hormone-sensitive**	26 (35.62)		
**Hormone-resistant**	31 (42.46)		
**Metastasis**	29 (39.72)		
**Death**	15 (20.54)		

## Data Availability

The data presented in this study are available in this article.
