# Correction: Bourefis et al. HSP27/Menin Expression as New Prognostic Serum Biomarkers of Prostate Cancer Aggressiveness Independent of PSA. *Cancers* 2022, *14*, 4773

**DOI:** 10.3390/cancers18060943

**Published:** 2026-03-13

**Authors:** Asma Bourefis, Hajira Berredjem, Omar Djeffal, Thi Khanh Le, Sophie Giusiano, Palma Rocchi

**Affiliations:** 1 Laboratory of Applied Biochemistry and Microbiology, Department of Biochemistry, Faculty of Sciences, Badji Mokhtar-Annaba University, Annaba 23000, Algeria; 2 Private Medical Uro-Chirurgical Cabinet, Annaba 23000, Algeria; 3 Predictive Oncology Laboratory, Centre de Recherche en Cancérologie de Marseille, Inserm UMR 1068, CNRS UMR 7258, Institut Paoli-Calmettes, Aix-Marseille University, 27 Bd. Leï Roure, 13273 Marseille, France; khanh.le-thi@inserm.fr; 4 Department of Pathology, Hôpital Nord, Chemin des Bourrely, Aix Marseille University, CEDEX 20, 13915 Marseille, France

## Text Correction

There was an oversight in the original publication [1]. A correction has been made to the Conflicts of Interest statement: The University of British Columbia has submitted patent applications on Apatorsen, an antisense inhibitor of Hsp27, listing P.R. as an inventor. This IP has been licenced to OncoGenex Technologies, a Vancouver-based biotechnology company. P.R. is a co-founder of SilonTx (https://silontx.com), a biotech company started in April 2024, focusing on precision medicine and nucleic acid therapeutics.

The authors state that the scientific conclusions are unaffected. This correction was approved by the Academic Editor. The original publication has also been updated.
